# Proteo-metabolomics reveals compensation between ischemic and non-injured contralateral kidneys after reperfusion

**DOI:** 10.1038/s41598-018-26804-8

**Published:** 2018-06-04

**Authors:** Honglei Huang, Leon F. A. van Dullemen, Mohammed Z. Akhtar, Maria-Letizia Lo Faro, Zhanru Yu, Alessandro Valli, Anthony Dona, Marie-Laëtitia Thézénas, Philip D. Charles, Roman Fischer, Maria Kaisar, Henri G. D. Leuvenink, Rutger J. Ploeg, Benedikt M. Kessler

**Affiliations:** 1 Oxford Transplant Centre, Nuffield Department of Surgical Sciences, University of Oxford, Oxford, UK; 2 Target Discovery Institute, Nuffield Department of Medicine, Old Road Campus, University of Oxford, Oxford, UK; 3 Department of Surgery, University Medical Center Groningen, Groningen, The Netherlands; 4 Department of Surgery and Cancer, Sir Alexander Fleming Building, Imperial College London, London, UK; 5 Cardiac Technology Centre, Kolling Institute of Medical Research, Royal North Shore Hospital, University of Sydney, Sydney, Australia; 6 Research and Development, NHS Blood and Transplant, Bristol, BS34 7QH UK

## Abstract

Ischaemia and reperfusion injury (IRI) is the leading cause of acute kidney injury (AKI), which contributes to high morbidity and mortality rates in a wide range of injuries as well as the development of chronic kidney disease. The cellular and molecular responses of the kidney to IRI are complex and not fully understood. Here, we used an integrated proteomic and metabolomic approach to investigate the effects of IRI on protein abundance and metabolite levels. Rat kidneys were subjected to 45 min of warm ischaemia followed by 4 h and 24 h reperfusion, with contralateral and separate healthy kidneys serving as controls. Kidney tissue proteomics after IRI revealed elevated proteins belonging to the acute phase response, coagulation and complement pathways, and fatty acid (FA) signalling. Metabolic changes were already evident after 4 h reperfusion and showed increased level of glycolysis, lipids and FAs, whilst mitochondrial function and ATP production was impaired after 24 h. This deficit was partially compensated for by the contralateral kidney. Such a metabolic balance counteracts for the developing energy deficit due to reduced mitochondrial function in the injured kidney.

## Introduction

Ischaemia and reperfusion injury (IRI) is characterized by a temporary restriction of blood flow to an organ followed by restoration of blood supply and re-oxygenation. Associated tissue injury may occur in the context of infarction, sepsis and organ transplantation^1^. In the kidney, IRI can lead to acute kidney injury (AKI), classified by elevated blood levels of urea nitrogen and creatinine due to impaired filtering capacity of the kidney^2^. Although the pathophysiology of IRI is not completely understood, several mechanisms resulting in AKI have been reported. During ischemia, the lack of oxygen and nutrition supply leads to ATP depletion and acidosis as a result of anaerobic metabolism with lactate overproduction^3–5^. ATP-dependent ion transport systems are impaired in ischaemic kidneys, leading to calcium accumulation, osmotic cell swelling and apoptotic or necrotic cell death. Upon reperfusion, restored levels of oxygen stimulate mitochondrial oxidative phosphorylation to produce ATP with the concurrence of harmful reactive oxygen species (ROS), which contributes to oxidative stress and lipid peroxidation^6^. ROS can then induce the release of inflammatory mediators and increase local leukocyte infiltration to ischaemic-injured sites that aggravates tissue injury^7^. One consequence is micro-vascular disruption that causes acute kidney failure and affects long-term graft survival^8–10^. Previous studies have examined various aspects of IRI in kidney injury^6,11,12^, including the use of proteomic and metabolomic profiling to discover renal biomarker candidates of kidney ischemic injury^4,13–16^. In particular, mitochondrial dysfunction and metabolic changes including lactate, succinate, choline, taurine and fatty acids have been observed after renal ischemic insults^5,17,18^. However, a more comprehensive study that integrates proteomic and metabolomic alterations at the initial stages of reperfusion after an ischemic insult has not yet been carried out in a more systematic fashion.

We used a non-lethal unilateral long IRI model in the rat^19^ and carried out an unbiased integrative proteo-metabolomic study in combination with mitochondrial function analysis of kidneys exposed to IRI to investigate its effects at the molecular level.

## Results

### Histological and apoptotic changes post IRI

**Figure 1:**
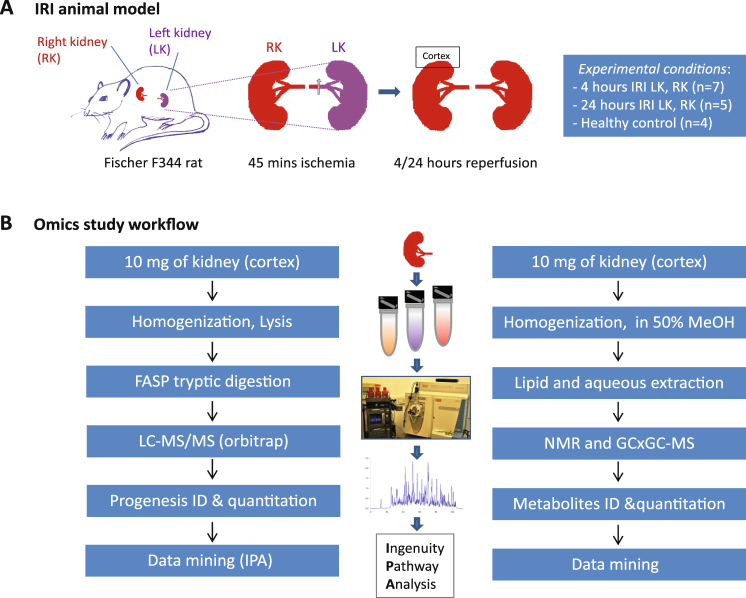
Experiment design and work flow of the proteome and metabolome study. (**A**) Ischaemia reperfusion Injury (IRI) animal model in male Fischer F344 rats. Ischaemia was induced for 45 min followed by 4 h (4 h-IRI; n = 7) or 24 h (24 h-IRI; n = 5) reperfusion. Contralateral kidneys served as endogenous controls (4 h-C, 24 h-C). In addition, kidneys removed from rats without IRI served as healthy controls (HC; n = 4). (**B**) Proteomic sample preparation, mass spectrometry analysis and data mining are outlined in the left panel. The middle panel presents the kidney tissue, equipment used for sample preparation, and bioinformatics tools for data analysis. Metabolomic sample preparation, ^1H^NMR analysis, and data mining are displayed in the right panel.

**Figure 2:**
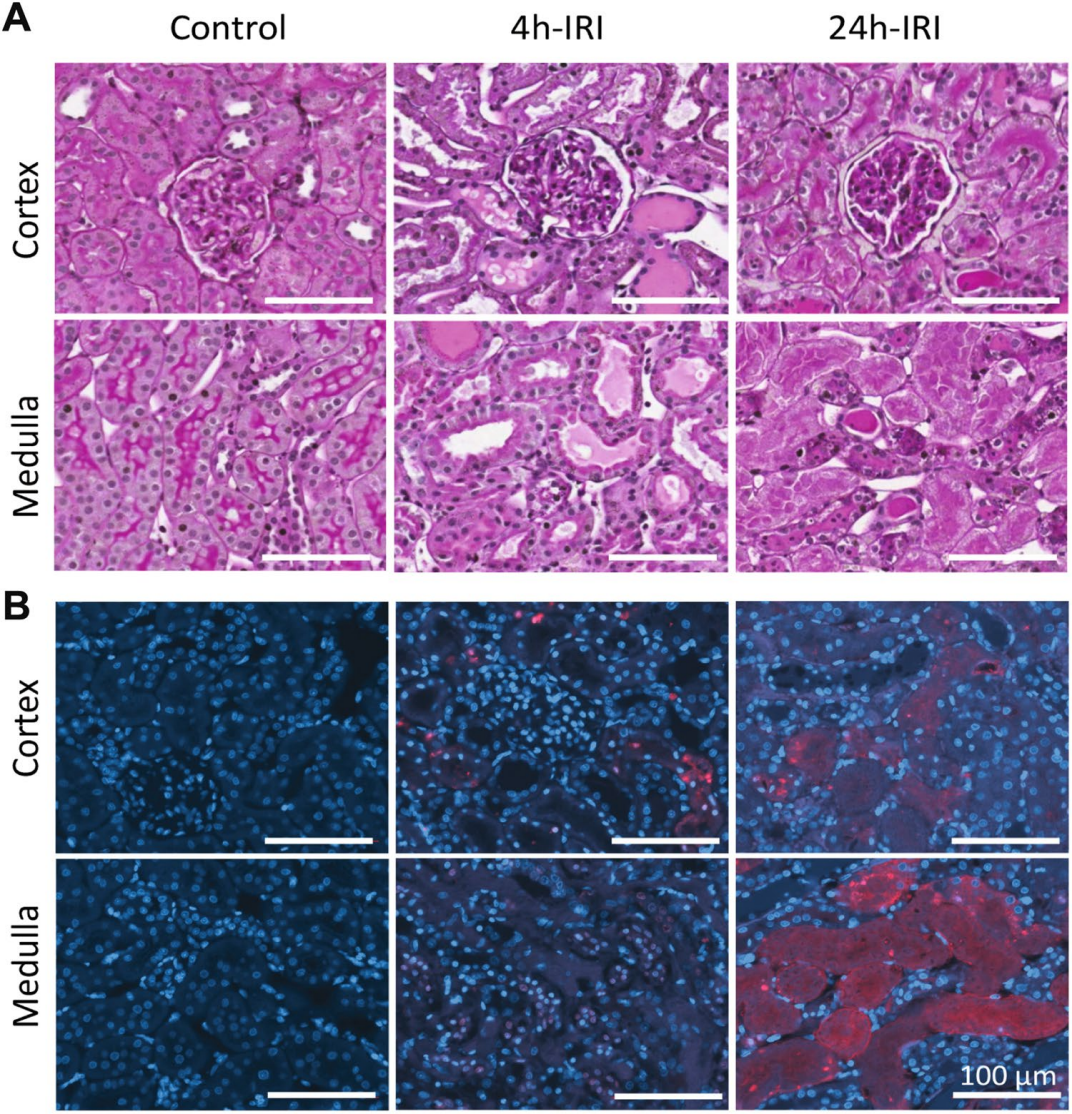
Histology and staining for apoptosis in cortex and medulla sections after IRI. (**A**) PAS staining for histological changes in 4 h-IRI, 24 h-IRI, and HC. Intratubular cast formation is evident in 4 h-IRI, while marked necrosis is observed in 24 h-IRI (**B**) Terminal Deoxynucleotidyl Transferase dUTP nick end labelling (TUNEL) staining for apoptosis in 4 h-IRI, 24 h-IRI, and HC. Break down DNA is shown in red, nuclei stained with DAPI are shown as blue and co localization is shown as pink. Apoptotic tubular cells were detected in 4 h-IRI in both cortex and medulla sections. Massive DNA break down in 24 h-IRI indicates extensive cell death.

Kidneys subjected to 45 min of warm ischaemia and 24 h of reperfusion (24 h-IRI) (Fig. 1) displayed severe injury and tubular necrosis, identified by the reduced number of tubular nuclei (Fig. 2A). This trait was not observed in 4 h-IRI or the control kidneys (4 h-C, 24 h-C, HC) (Figure S1). With TUNEL staining we could detect tubular apoptosis as early as 4 h post-injury in both cortex and medulla of the kidneys subjected to ischaemia (4 h-IRI), eventually resulting in necrosis after 24 h (24 h-IRI) (Fig. 2B). This was consistent with the level of injury observed in the histological sections of the same area stained with PAS.

### Proteomic analysis after IRI

Having confirmed tissue injury, kidney cortex samples of 4 h-IRI (n = 5, LK), 4 h-C (n = 5, RK), 24 h-IRI (n = 5, LK), 24 h-C (n = 5, RK) and HC (n = 4) were subsequently analysed by LC-MS/MS. In total, 1,055,427 MS/MS-spectra were acquired, resulting in the identification of 2,798 proteins with an estimated false discovery rate (FDR) of 0.96%.

**Figure 3:**
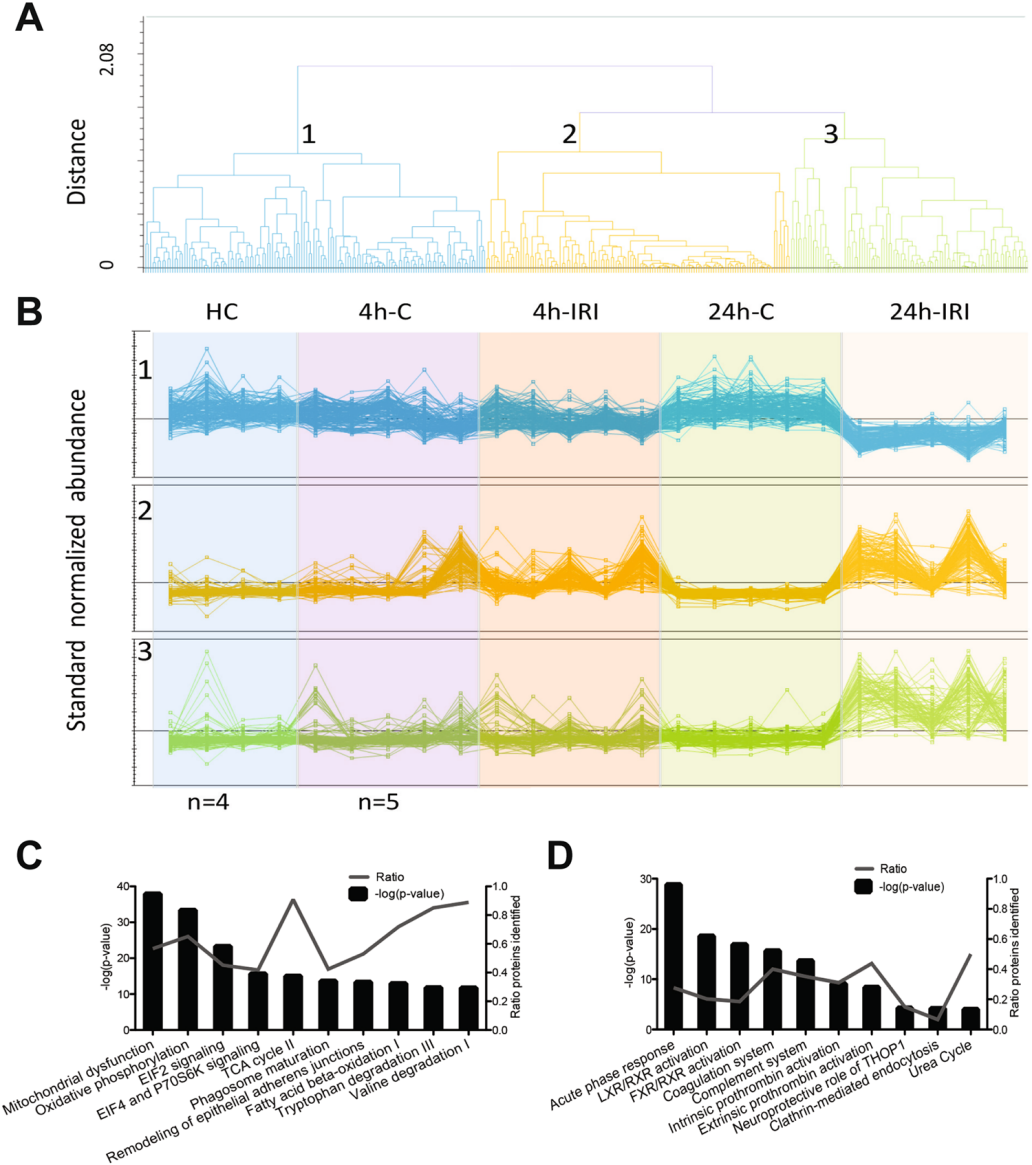
Hierarchical clustering and canonical pathway analysis of the IRI kidney proteome. (**A**) From all 2798 proteins identified, 363 proteins with a significant change in abundance (ANOVA p < =0.05) between 24 h IRI and 24 h-C were clustered into three groups (1, 2, 3) based on their protein abundance pattern in 4 h-IRI, 24 h-IRI, their endogenous and healthy controls. (**B**) Abundance profiles of the 363 proteins binned into the three clusters. Cluster 1 shows downregulation of 140 proteins in 24 h-IRI vs other experimental groups. Cluster 2 shows 125 upregulated proteins in 4 h-IRI and 24 h-IRI vs other experimental groups. Cluster 3 shows 98 upregulated proteins in the 24 h/4 h-IRI only. (**C**) Top 11 canonical pathways revealed by the analysis of all identified 2798 proteins from 4 h IRI/C, 24 h IRI/C and HC. The -log (p-value) for pathway activation is displayed on the left Y-axis, the percentage of identified protein members for each pathway is shown on the right Y-axis. (**D**) Canonical pathway analysis of the 363 significantly and differentially expressed proteins between 24 h IRI and C. There was no overlap between these top 11 pathways (**D**) and the top 11 pathways described in (**C**), indicating enrichment of pathways relevant to alterations of the kidney proteome after IRI. 4 h-IRI and 24 h-IRI: kidneys subjected to IRI; 4 h-C and 24 h-C: contralateral controls; HC: healthy controls.

Four hours post IRI, 55 proteins were found to be differently expressed with ≥2-fold change and a p < 0.05 between 4 h-IRI, 4 h-C, and HC (Table S1). After 24 h reperfusion, 397 proteins were found to be different between 24 h-IRI and 24 h-C kidneys (Table S2). Subsequently, these proteins were clustered and sorted according to their abundance patterns. Using hierarchical clustering analysis, we assigned 363 proteins to three groups based on their unique abundance patterns (Fig. 3A, Table S2). Among these 363 differentially expressed proteins, 140 were decreased in 24 h-IRI compared to other groups (Fig. 3B, upper panel) and 125 proteins were increased in both 4 h-IRI and 24h-IRI compared to 4 h-C, 24 h-C, and HC (Fig. 3B, middle panel). Ninety-eight proteins were increased only in 24 h-IRI kidneys compared to other groups (Fig. 3B, lower panel).

Ingenuity Pathway Analysis was applied to classify these proteins according to their function. Figure 3C shows the top 11 canonical pathways assigned for all identified proteins (2798). Assignment of the subset of 363 selected proteins revealed enrichment of unique pathways after IRI, including acute phase response signalling, coagulation and complement pathways, and liver X and retinoid X receptor (LXR/RXR) activation (Fig. 3D).

### IRI induces stress, coagulation and complement pathways and fatty acid signalling in kidneys

**Figure 4:**
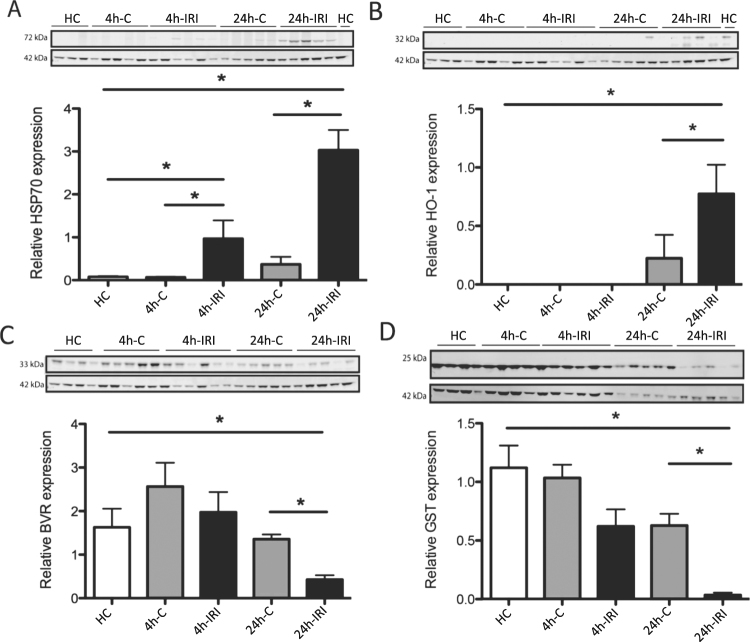
Western blot validation of stress proteins and antioxidants. (**A**) There was an increased level of HSP70 in both 4 h-IRI and 24 h-IRI compared to their endogenous contralateral kidneys (4 h-C, 24 h-C) and healthy controls (HC). (**B**) Increased level of HO-1 in 24 h-IRI compared to 24 h-C and HC. (**C**) Reduced level of BVR in 24 h-IRI compared to 24 h-C and HC. (**D**) The level of GST was significantly reduced in 24 h-IRI compared to 24 h-C and HC. Protein level was normalised to β-actin abundance. Data is presented as mean ± SEM. ^*^p-value < 0.05.

Proteins differentially expressed as listed in Tables S1 and S2 included stress proteins. Heat Shock Protein 70 (HSP70 or HSPA1A) and Heme-oxygenase 1 (HO-1) were significantly upregulated after IRI which was validated using Western blot analysis (Fig. 4A,B). To validate if our IRI model induced ROS production, we measured the abundance of the antioxidants Glutathione S-transferase (GST) and Biliverdin Reductase A (BVR) in the kidney. Both GST and BVR protein levels were significantly decreased in 24 h-IRI compared to 24 h-C and HC.

The complement and coagulation pathway was highly upregulated after IRI, possibly through activation or post-injury leucocytosis. Most of the proteins did not change after 4 h IRI, but showed significant increases at 24 h post IRI (Figure S2A, Table S2). C4 was further validated by Western blot, confirming its increased levels post-IRI (Figure S2B).

**Figure 5:**
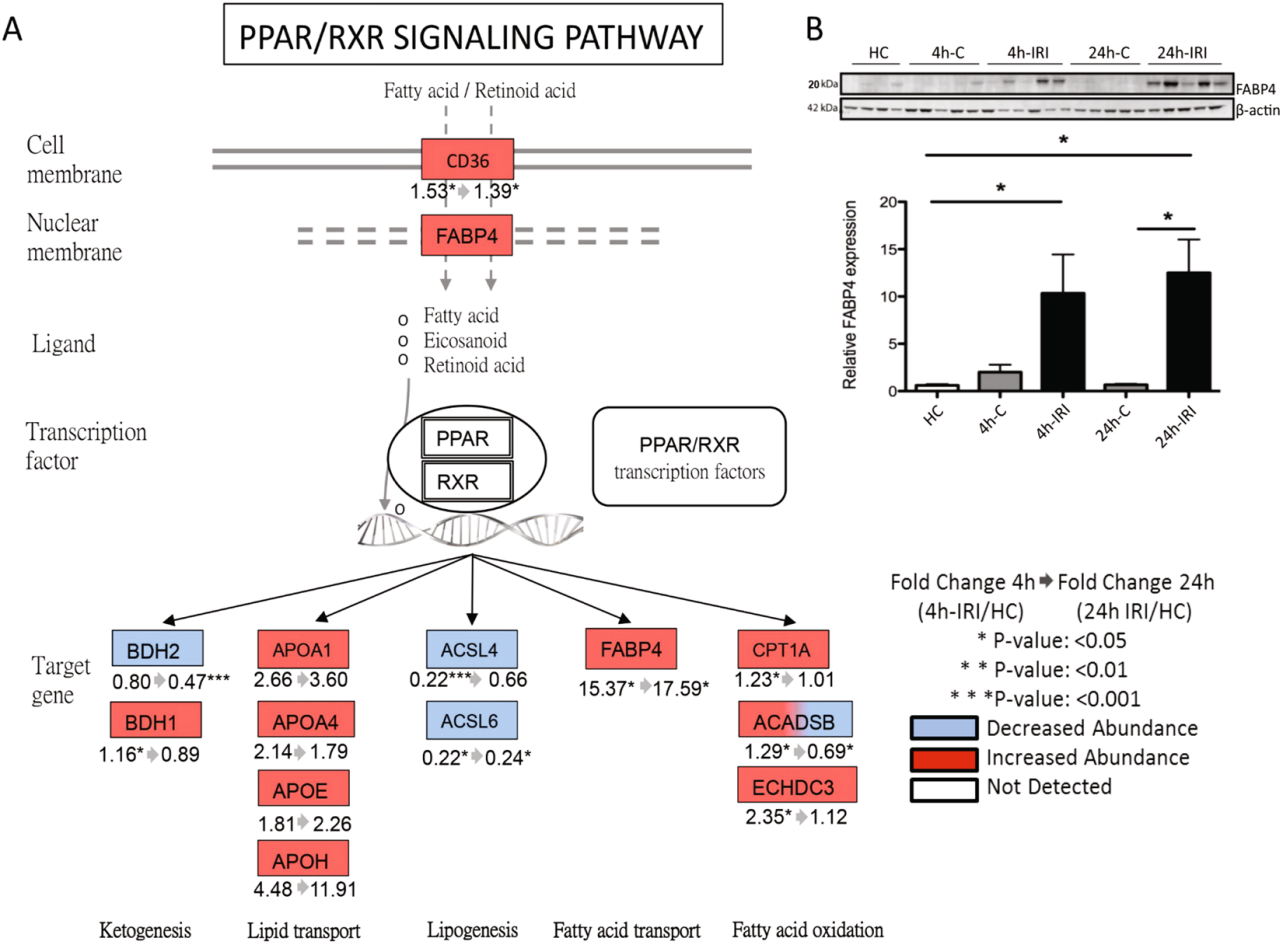
Peroxisome proliferator-activated receptor (PPAR)/retinoid X receptor (RXR) pathway activation post IRI. (**A**) PPAR is a type II nuclear receptor that includes the liver X receptor (LXR) and dimerizes with RXR to form LXR/RXR. LXR/RXR can be activated by fatty acids and its derivatives. CD36 and FABP4 are proteins that transport fatty acids and retinoid acids across the cell- or nuclear membranes. Upon activation, LXR/RXR acts as a transcription factor and thus plays a role in metabolism and clearance of lipids. In our study, proteins involved in fatty acid oxidation (CPT1A, ACADSB, and ECHDC3) were increased in 4 h-IRI but restored to normal levels after 24 h reperfusion. FABP4 was elevated in 4 h-IRI and 24 h-IRI. Lipid transporters (APOA1, APOA4, APOE, and APOH) were also increased in both 4 h-IRI and 24 h-IRI, but did not reach statistical significance. Ketone body generating protein BDH1 was increased in 4 h-IRI, while BDH2 was reduced at 24 h-IRI. Proteins involved in fatty acid synthesis (ACSL4, ACSL6) were reduced in both 4 h-IRI and 24-IRI. (**B**) Increased abundance of FABP4 in 4 h-IRI and 24 h-IRI compared to 4 h-C, 24h-C, and HC measured by Western blot. Data is presented as mean ± SEM. 4 h-IRI and 24 h-IRI: kidneys subjected to IRI; 4 h-C and 24 h-C: contralateral controls; HC: healthy controls.

LXR/RXR signalling is part of the nuclear receptor family that interacts with peroxisome proliferator-activated receptor (PPAR). Thirteen proteins identified in our proteome data were assigned to this pathway (Fig. 5A) including significantly increased levels of upstream regulator fatty acid transporter CD36 and FABP4 in both 4 h-IRI and 24 h-IRI. The increased abundance of FABP4 measured by proteomics was validated by Western blot (Fig. 5B). Other downstream proteins were also changed. The fatty acid (FA) oxidation proteins CPT1A, ACADSB, and ECHDC3 were significantly increased in 4 h-IRI but, except for ACADSB, returned to normal levels after 24 h (24 h-IRI). Proteins involved in lipid transport (APOA1, APOA4, APOE, and APOH) were increased in both 4 h-IRI and 24 h-IRI kidneys, but changes did not reach statistical significance. Proteins involved in ketogenesis were affected. BDH1 was significantly upregulated in 4 h-IRI, while BDH2 was significantly downregulated in both 4 h-IRI and 24h-IRI. In addition, we found proteins involved in lipogenesis (ACSL4 and ACSL6) to be significantly downregulated in both 4 h-IRI and 24 h-IRI as compared to the endogenous controls (4 h-C, 24 h-C).

### Metabolomics changes after IRI

**Figure 6:**
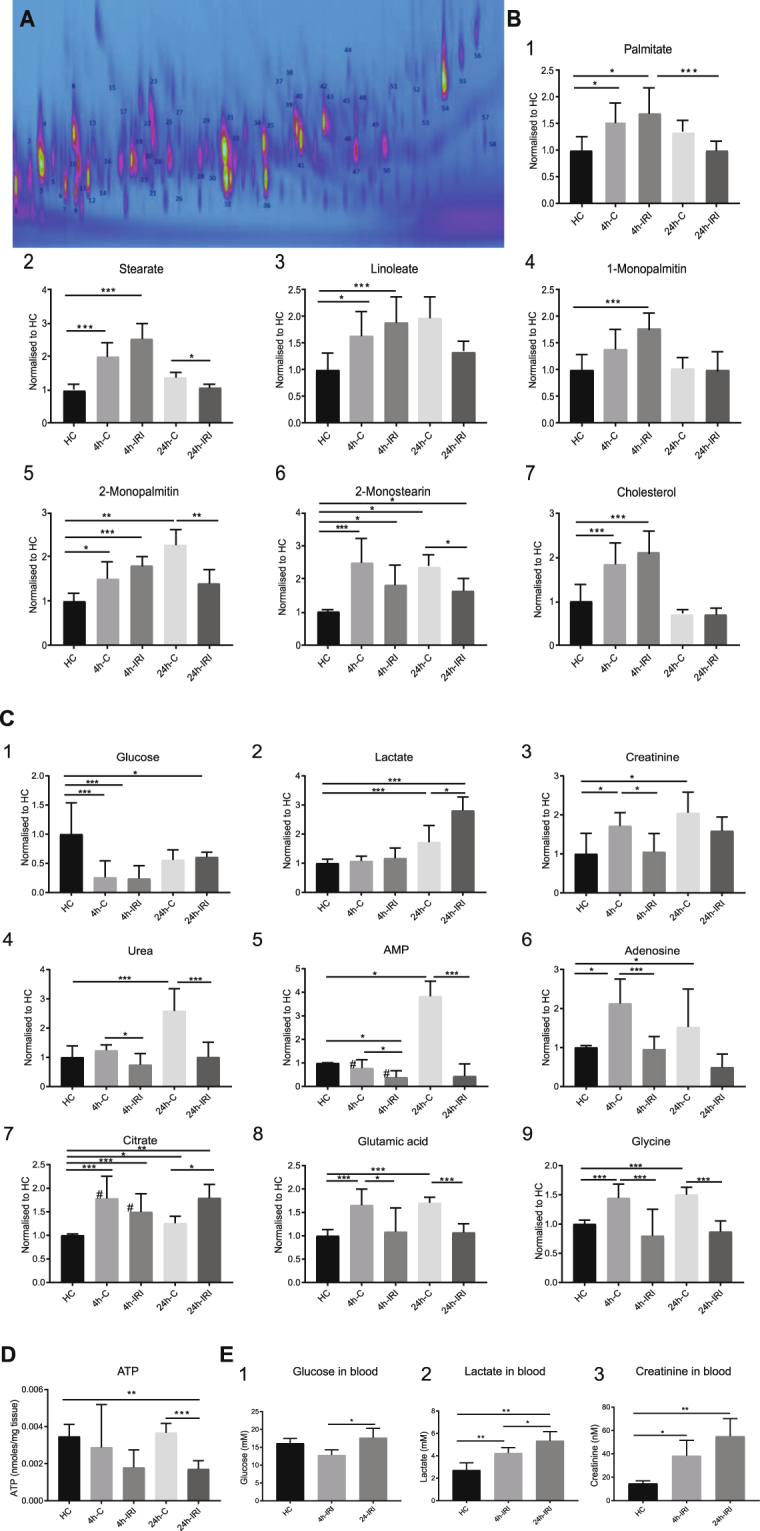
Metabolites quantified by NMR and GCxGC-MS in both tissue and plasma after IRI. (**A**) Extracted metabolites were analysed by GCxGC-qMS. A representative 2D-GCxGC map is shown in which the identified metabolites are numbered. (**B**) Relative quantities of 1. Palmitate, 2. Stearate, 3. Linoleate, 4. 1-monopalmitin, 5. 2-monopalmitin, 6. 2-monostearin, and 7. Cholesterol detected in control (HC), contralateral (4 h-C, 24 h-C and injured kidney tissue (4 h-IRI, 24 h-IRI). (**C**) Relative quantities of 1. Glucose, 2. Lactate, 3. Creatinine, 4. Urea, 5. AMP, 6. Adenosine, 7. Citrate, 8. Glutamic acid and 9. Glycine detected in control (HC), contralateral (4 h-C, 24 h-C and injured kidney tissue (4 h-IRI, 24 h-IRI). Metabolite levels are provided as detected by GCxGC-qMS, and the values labelled with (#) by ^1^H-NMR. (**D**) ATP levels were quantified using a luminescent ATP detection assay kit in kidney tissues (normalized to tissue weight). (**E**) Metabolites measured using conventional clinical measurement in plasma of HC, 4 h and 24 IRI animals: 1. Glucose, 2. Lactate and 3. Creatinine. Data is presented as mean ± SEM. 4 h-IRI and 24 h-IRI: kidneys subjected to IRI; 4 h-C and 24 h-C: contralateral controls; HC: healthy controls. ^*^p-value < 0.05, ^**^p-value < 0.01, ^***^p-value < 0.001.

**Figure 7:**
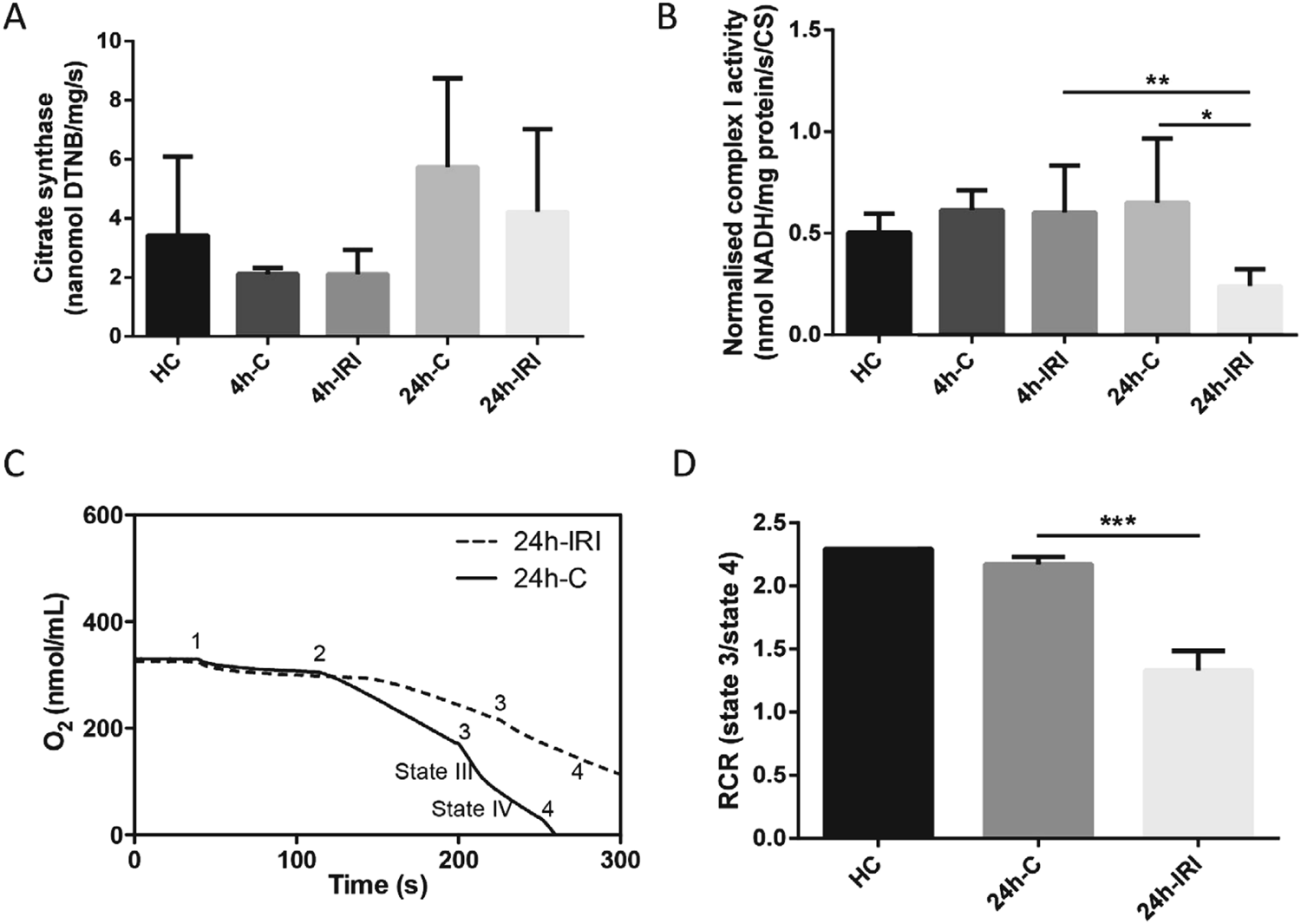
Mitochondrial complex activity and oxygen consumption analysis after IRI. (**A**) Citrate synthase activity was used for normalization and measured in 4 h-IRI and 4 h-C (n = 7), 24 h-IRI and 24 h-C (n = 4), and HC (n = 4). (**B**) Complex I activity was measured in the same subset of samples and normalized to citrate synthase. Significant reduction of normalized complex I activity was observed in 24 h-IRI compared to 24 h-C and 4 h-IRI. (**C**) The respiration states were measured by oxygen consumption. State I: after adding mitochondria (1). State II was recorded by addition of succinate (2). State III-IV by addition of ADP (3), and state V by adding FCCP (5). Kidneys form 24 h-IRI showed a significantly impaired oxygen consumption rate. (**D**) Respiratory control ratio (RCR) was measured in 24 h-IRI (n = 3), 24 h-C (N = 3), and a HC (n = 1) by comparing state III and -IV oxygen consumption levels. There is a significant RCR reduction in 24 h-IRI compared to 24 h-C. Data is expressed as mean±SD. ^*^p-value < 0.05, ^**^p-value < 0.01, ^***^p-value < 0.001.

After IRI, it can be expected to find rapid metabolomic changes. Therefore, we performed a metabolomic study to further explore these effects by using GCxGC-qMS and NMR on 4 hours (n = 7, LK), 4 h-C (n = 7, RK) and 24 hours post IRI (n = 5, LK), 4 h-C (n = 5, RK), and HC (n = 2 for 4 h, and n = 2 for 24 hours) kidney samples. Using a previously established GCxGC-qMS method^20^, we identified 627 molecular features (blobs) in kidney tissue, from which 58 unique metabolites (~10%, Fig. 6A) were identified with a confidence score of >80 (maximal 100, Table S3). This was complemented by ^1^H-NMR analysis revealing 29 unique metabolites, giving rise to a total of 71 (Table S3). GCxGC-qMS and ^1^H-NMR were used to quantify 44 of them across all conditions, and for those metabolites detected with both methods, we observed comparable relative abundance values (Table S4). Lipid- and FA metabolites such as palmitate (Fig. 6B-1), stearate (Fig. 6B-2), linoleate (Fig. 6B-3), 1-monopalmitin (Fig. 6B-4), 2-monopalmitin (Fig. 6B-5), 2-monostearin (Fig. 6B-6), and cholesterol (Fig. 6B-7) appeared to accumulate after 4 h followed by a reduction after 24 h IRI (Table S4). In addition, we noted reduced glucose levels in both 4 h-IRI and 4 h-C kidneys that were sustained in 24 h-IRI as compared to HC (Fig. 6C-1). Interestingly, glucose levels were essentially unaltered in plasma of 4 h and 24 h-operated animals (Fig. 6E-1). Lactate levels, however, were increased in 24 h-IRI as compared to HC (Fig. 6C-2). Interestingly, lactate levels were also significantly elevated in the plasma of 4 h-operated animals and even to a greater extent after 24 h (Fig. 6E-2). Similarly, blood creatinine levels were higher in both 4 h and 24 h post IRI (Fig. 6E-3), suggesting a general inability to clear these metabolites within this period after IRI.

Remarkably, we observed compensatory changes in metabolite levels in the uninjured organ of animals subjected to kidney IRI, in particular after 24 reperfusion. For instance, a strong elevation of urea and AMP in contralateral kidneys after 24 h post IRI, which was not observed in the injured kidney counterpart (Fig. 6C-4, 6C-5). In addition, adenosine, glutamic acid and glycine levels were increased in a more prominent fashion in contralateral kidneys, particularly after 24 h (Fig. 6C-6–C-9).

**Figure 8:**
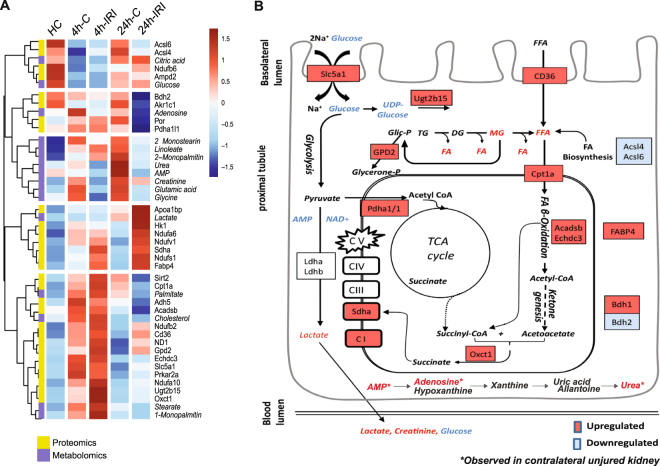
Integrated proteomic and metabolomic analysis 4 h and 24 h post IRI. (**A**) Metabolites and proteins altered during IRI were shortlisted based on their changed abundance in 4 h-IRI and 24 h-IRI compared to 4 h-C, 24 h-C and HC. A heat map using both metabolites (Italic font-style) and proteins (normal font-style) indicates a distinct pattern, in particular for FA β-oxidation and ketogenesis. (**B**) Proposed model of energy homeostasis 4 h and 24 h post IRI. Increased FFA metabolism was suggested according to increased FA transporters (CD36, Cpt1a), monoglyceride levels, FA β-oxidation enzymes (Acadsb, Echdc3), and ketogenesis. The metabolites derived from FA β-oxidation (acetyl-CoA) can directly feed into and sustain the TCA cycle. Metabolite generated from the TCA cycle (succinyl-CoA) and products from ketogenesis (acetoacetate) can subsequently form succinate catalysed by Succinyl-CoA:3-Ketoacid-CoA Transferase (Oxct1). Succinate is an essential substrate that can be fed directly into the electron transport chain (Complex II) and into the TCA cycle, maintaining and providing essential mitochondrial substrates. Accumulation of AMP and urea was observed in contralateral (uninjured) kidneys. Statistical analysis using a non-parametric Mann-Whitney test was used to assess significant changes in metabolite and protein abundances (Table S4).

### Impaired mitochondrial function 24 h after IRI

The TCA cycle intermediate citrate (Fig 6C-7) appeared to be elevated in all conditions as compared to control (Table S3). Consistent with previous reports, we found that ATP levels were significantly decreased in 24 h–IRI kidney tissue as compared to 24 h-C and HC (Fig. 6D). These observations indicated altered function of mitochondria, which are the main ATP-producing source under aerobic conditions. To test this, we used a mitochondria activity assay, in which citrate synthase activity was used for normalisation (Fig. 7A). Normalised complex I activity was significantly reduced in 24 h-IRI compared to 24 h-C or 4 h-IRI kidneys (Fig. 7B). Mitochondrial complex II-III and -IV activities were also measured in 4 h-IRI, 4 h-C, 24 h-IRI, 24 h-C, and HC but were not significantly changed (Figure S3).

Since mitochondrial complex I activity was impaired at 24 h-IRI, we also measured the mitochondrial oxygen consumption and RCR using mitochondria extracted from 24 h-IRI (n = 3), 24 h-C (n = 3), and a HC (n = 1) (Fig. 7C,D). The RCR and oxygen consumption were significantly decreased in 24 h-IRI as compared to 24 h-C.

### Proteo-metabolic integrated analysis reveals IRI-induced β-oxidation and compensatory effects in uninjured kidneys

To provide a better understanding of the altered metabolites and proteins in our data, we applied an integrative proteo-metabolomic analysis^21^, in which we included metabolites and corresponding enzymes that showed statistical differences between 4 h-IRI, 24 h-IRI, 4 h-C, 24 h-C or HC. Sixteen metabolites belonging to the lipid-, glycolysis-, and TCA cycle pathways, and 29 metabolic-related proteins with a p < 0.05 in at least one comparison were selected and displayed in a heat map (Fig. 8A, Table S4) and summarising scheme (Fig. 8B). Hierarchical cluster analysis revealed the existence of five phenotypes: i) Decreased substrates in 4 h-IRI and/or 4 h-C compared to HC and 24 h-IRI/C. This includes proteins involved in FA biosynthesis (Acsl6, Acsl4), metabolites involved in energy metabolism (glucose and citric acid); ii) Decreased substrates prevalently in 24 h-IRI compared to the other conditions. This group comprises adenosine, proteins involved oxidation and reduction reactions (Por) and enzyme that play role in the TCA cycle (Pdha1/1). iii) Metabolites increased prevalently in 24 h-C animals as compared to the other conditions. This includes FFAs (2-Monostearin, 2-Monopalmitin and linoleate), non-essential amino acids (Glutamic acid, glycine), urea, AMP and creatinine. iv) Proteins and metabolites prevalently increased in 24 h-IRI including enzymes involved in oxidative phosphorylation (Ndufa6, Ndufv1, and Ndufs1), fatty acid binding protein (Fabp4), glycolysis enzyme (Hk1) and lactate. v) Enzymes and metabolites elevated in 4 h-IRI and 4 h-C such as glucose transporter (Slc5a1), FA transporter (CD36), components of oxidative phosphorylation (ND-1), detoxification enzymes (Adh5, Ugt2b15), mitochondrial biogenesis (Sirt2), FFA metabolism (Cpt1a, Acadsb, Echdc3, palmitate, stearate, 1-monopalmitin) and ketone metabolism (Oxct1).

## Discussion

This study has attempted to evaluate effects of renal IRI at the protein and metabolite levels with the aim to identify pathways for potential intervention. Unilateral renal ischemia reperfusion is a robust model for both acute and chronic kidney injury^22^. A 45 min ischemia model is commonly applied, as it allows the introduction of significant injury without causing animal death. To avoid the unnecessary use of sham animals and to assess more systemic effects after IRI, contralateral (right) kidneys served as controls to reduce variation between operation procedure and animals. As a baseline, we used four additional healthy controls to define baseline protein expression levels. Ischemia results in a metabolic imbalance. One hallmark is succinate accumulation, and upon reperfusion, accumulated succinate contributes to ROS production through the mitochondrial complex II (17), consistent with our observations at 4 h and 24 h reperfusion after ischemia. We observed an increased abundance of stress proteins (HSP70, HO-1) (Fig. 4), reduced (consumed) levels of antioxidants (BVR, GST) (Fig. 4) and severe tubular injury (Fig. 2), indicative of a strong response to oxidative stress. Oxidative stress can activate the nuclear factor erythroid 2-related factor 2 (Nrf2) pathway that induces downstream antioxidant gene transcription such as GST and HO-1^23,24^.

It is known that the coagulation and complement systems become activated post IRI in the deceased kidney donor^25,26^. Coagulation plays a critical role in homeostasis but can also further activate the complement system^27,28^. Consistent with the literature, we observed a strong upregulation of these pathways including enhanced abundance of C3 and C4 and their respective downstream products, C5, C6, C8, and C9 after 24 h IRI. Complement induces renal injury post IRI and inhibition can prevent these detrimental effects^29,30^. This treatment option is currently being explored in the human transplant setting^31,32^.

Most remarkably, proteomics analysis revealed that free fatty acid (FFA) signalling through LXR and PPAR had changed after IRI (Figs 3D and 5). LXR and PPAR are nuclear receptors involved in regulating nutrient metabolism^33,34^, in particular FA synthesis (LXR) and catabolism (PPAR)^35^. Downstream transcription targets of PPARγ signalling are genes involved in lipid metabolism^36^, which prompted us to further explore this phenomenon in IRI. In our data, we observed a trend of FFA levels increasing between HC, 4 h-C and 4 h-IRI. Notably, after 24 h, this effect was less evident in 24 h-IRI. This might suggest accelerated FFA utilization through β-oxidation feeding into TCA cycle that led to their depletion. It is known that FFA levels decrease after ischaemia^37^, but reperfusion injury is correlated with a persistent elevation of FFAs^36^. The mechanism of elevated FFAs post IRI remains unclear, but could be the result of increased uptake from exogenous sources, stimulation of intercellular production (by lipases or *de-novo* synthesis) or the failure of cells to reutilize or dispose FFAs (β-oxidation). Reduced levels of enzymes involved in FFA biosynthesis enzymes, Acsl4 and Acsl6, are incompatible with intercellular *de-novo* synthesis. Increased levels of enzymes in the β-oxidation process suggested elevated FFA utilization, possibly promoted further by greater FFA plasma levels observed in animal models after IRI^5,22^. Indeed, upregulated CD36 levels suggest accelerated cellular uptake from the systemic circulation. Next to being used as an energy source, internalized FFAs can be delivered to the nucleus by FFA transport proteins, such as FABP4, that also appears elevated (Fig. 5), affecting gene transcription of enzymes in lipid metabolism through the PPAR pathway^38^. Integrative proteo-metabolomic analysis showed increased FFA β-oxidation after IRI, suggesting FFA catabolism through β-oxidation to produce ATP. This was supported by a study demonstrating that proximal tubules use FFAs as preferential energy resource as it has limited glucose metabolism enzyme activity, in contrast to what is observed in the distal tubular compartment^39^. FFA β-oxidation occurs in the mitochondrial compartment, which may explain why the FFA transport protein Cpt1a was also increased after IRI.

IRI can induce anaplerotic reactions to compensate for a deficiency of metabolic intermediates^4^. The product of FA β-oxidation, Acetyl-CoA, can either feed the TCA cycle or contribute to ketone genesis. We observed an increase in ketogenesis, reflected by a greater level of 3-hydroxybutyrate dehydrogenase (Bdh1). In addition, acetoacetate, as a product of ketogenesis, can react with the TCA cycle derived succinyl-CoA and form succinate through the activity of Oxct1. Our results found that both Bdh1 and Oxct1 were increased post IRI, suggesting utilization of ketones and thereby providing an alternative source for succinate. Succinate can also be utilized directly by mitochondrial complex II (Sdha) for electron transport. In the IRI kidney, increased levels of Sdha might suggest an induction of the electron transporters in the mitochondria. In line with this, enzymes belonging to mitochondrial complex I (Ndufa10, Ndufb2, Ndufb6, Ndufv1, Ndufa6, and Ndufs1) were found to be elevated in our IRI model.

IRI is an energy consuming process and is associated with impaired glucose metabolism^3^, mitochondrial injury^40,41^, and ATP depletion^6,42^. In our study, glucose levels were significantly decreased in tissue at 4 h-IRI and 24 h-IRI, although tissue ATP content and mitochondrial function at 4 h-IRI were not changed. This may be compensated by an increased level of the glucose transport protein Slc5a1 and an increased anaerobic activity with elevated lactate levels in blood and tissue. However, ATP levels, mitochondrial complex I activity, and oxygen consumption could not be sustained and were significantly decreased in 24 h-IRI kidneys (Fig. 7). It is evident that mitochondrial dysfunction resulted in a significant energy shortage as reflected by decreased levels of AMP.

Strikingly, we noted the greatest metabolic effects after 24 hours in the contralateral kidney, where elevated levels of urea and AMP were detected. Urea cycle transforms toxic ammonia into urea for excretion. This metabolism requires two inorganic phosphates, removed from one molecule of ATP, to produce AMP and Urea. If not re-phosphorylated to ATP or removed by circulation, AMP is catabolised through hypoxanthine-xanthine-uric acid formation and subsequent elimination (Fig. 8B)^43,44^. Accumulation of these metabolites in the contralateral kidney is most likely because the injured kidney is functioning in a suboptimal fashion, consistent with a generally reduced creatinine clearance from the blood (Fig. 6E-3). The contralateral kidney is therefore taking up the functions of both kidneys.

In summary, this study has profiled the proteo-metabolomic landscape of IRI in kidney tissue and has revealed possible compensation mechanisms taken up by contralateral kidneys for the acute energy deficit after an ischemic insult (injured kidney). A clinically relevant consequence could therefore be the optimisation or protection of kidney metabolism, maximising substrate utilisation and ATP production as a strategy to ameliorate AKI.

## Methods

### IRI animal model

A rat model of ischaemia reperfusion injury was carried out using Fisher rats (F344) weighing 250–300 g. This study was approved by the Animal Welfare Ethics Review Board, and carried out in accordance with Home Office guidance on Operation of Animals (PPL 30/2750). A midline laparotomy was performed with dissection of the right and left renal pedicles. The left renal artery was clamped for 45 min inducing warm ischaemia (IRI). The contralateral right kidney served as an endogenous control (C) and remained untouched.

To evaluate short- and longer-term effects, rats were sacrificed after 4 h and 24 h reperfusion, respectively, and both the left (IRI-4 h; IRI-24 h) and right (C-4 h; C-24 h) kidneys were perfused with 30 ml of cold phosphate-buffered saline, then retrieved for further analysis. Kidneys from healthy control (HC) rats were also included as a base line to discriminate between local injury and systemic effects. Hence, the following groups of kidneys were included in this experiment: IRI-4 h (n = 7) and C-4 h (n = 7); IRI-24 h (n = 5) and C-24 h (n = 5); HC (n = 4). We have used a power calculation to determine the minimum number of samples (biological replicates) per group, taken into account that the rat rodent model is of congenic nature and therefore exerts much less genetic variability as compared to human subjects. We applied the following parameters: for a power of 0.8, a confidence of 0.05 and assuming a total variability of 25%, a fold-change of 1.5 and above can be measured reliably when at least four biological replicates per group are included in the analysis^45^.

### Histology and apoptosis staining

Dissected kidneys from 4 h-IRI (n = 7), 4 h-C (n = 7), 24 h-IRI (n = 5), 24 h-C (n = 5), and HC (n = 4) kidneys were fixed in 10% formalin, paraffin embedded, and sectioned (5μm). Periodic Acid Schiff (PAS) staining was processed as standard protocol^46^. Apoptosis analysis was performed by detecting endonucleolytic cleavage of chromatin (ApopTag, Merck Millipore, UK).

### Protein extraction, identification, and quantitation using mass spectrometry

The cortex tissue (outer portion, more red and granular consistency) was dissected (20–30 mg sections) from 4 h-IRI (n = 5), 4 h-C (n = 5), 24 h-IRI (n = 5), 24 h-C (n = 5), and HC (n = 4) kidneys under careful visual inspection. Tissue material was processed for analysis by mass spectrometry as described in detail in the Supplementary Information section (Fig. 1).

LC-MS/MS analysis was carried out by Nano-ultra performance liquid chromatography tandem mass spectrometry analysis as described in detail in Supplementary Methods.

### Western blot validation of differentially expressed proteins

Ten milligram of renal cortex was dissected and lysed in RIPA buffer containing protease inhibitors (Roche, USA). Western blot analysis was performed on 4 h-IRI (n = 7), 4 h-C (n = 7), 24 h-IRI (n = 5), 24 h-C (n = 5), and HC (n = 4) kidneys by loading 15 µg of proteins on 8–12% pre-cast Bis-Tris gels (Bio-Rad, USA) and transferred to PVDF membranes (Merck Millipore, USA). Membranes were incubated with rabbit anti-rat C4 (25 ug/ml), HSP70 (1:1000), GST (1:3000), FABP4 (1 mg/ml), BVR (1:5000) (Abcam, UK), and TLR4 (1:250) (Santa Cruz, USA) antibodies. β-actin was used as loading control (1:25,000) (Sigma, Germany). Dye-800-conjugated secondary antibodies were applied and visualised with an Odyssey Clx (Li-Cor, USA).

### Extraction of metabolites from kidney tissue and analysis using nuclear magnetic resonance spectroscopy (NMR) or gas chromatography mass spectrometry (GCxGC-MS)

Fifty micrograms of cortex tissues from 4 h-IRI (n = 7), 4 h-C (n = 7), 24 h-IRI (n = 5), 24 h-C (n = 5), and HC (n = 4) kidneys were placed in beads-beater tubes containing 1.5 ml of pre-chilled methanol/water (1:1), loaded onto a beads beater (Stretton, UK), and homogenised. Organic extracted metabolites were stored at −80 °C until analysis (Fig. 1). For more details, see Supplementary Methods.

Metabolite identification was performed by ^1^H-NMR using a Bruker Avance 600 MHz nuclear magnetic resonance spectrometer instrument, data sets were imported into MATLAB 7.0 software (MathWorks, USA) and assignments of endogenous metabolites were made by reference to published literature data^47^ and the in-house and online databases^48,49^. Multivariate statistical analysis was performed using SIMCA-P 13.0.

In addition, 2 µl of derivatized metabolites were injected into a GCxGC-qMS instrument (GP2010, Shimadzu) for a targeted analysis of a panel of molecules including FA’s, glucose and lactate as described previously^20^. Metabolites were identified by matching of collected fragment ions to the NIST library using GC solution software (v2.32). Metabolite quantitation was performed using Chrome square software v2.1.6 (Shimadzu, Japan). Metabolite quantity was normalized against myristic acid spiked in the samples for comparison.

### Plasma glucose, lactate, and creatinine measurements

Plasma samples from all animals were collected at 4 h and 24 h post IRI as well as in HC rats. Plasma samples were diluted (1:2) in PBS and glucose, lactate, and creatinine were measured using a Roche/Hitachi Modular System (Roche Diagnostics, The Netherlands).

### Integrative analysis of proteome and metabolome data

All the proteins/enzymes involved in energy metabolism were shortlisted based on altered metabolome profiles between 4 h-IRI, 4 h-C, and HC. Protein expression regulated by metabolites was also included from the proteome data. A total of 125 proteins were selected in the analysis, from which 29 were shortlisted based on their statistical significance and altered abundance (Table S4). From all measured metabolites, we selected a subset of 16 metabolites that were found to be significantly changed. We used R-based clustering of proteins and metabolites to visualise subgroups with different patterns of abundance (Fig. 8). A non-parametric Mann-Whitney test was applied to determine the significance of differences observed for proteins and metabolites.

An extensive description of the Material and Methods is available online in the Supplementary Information section.

## Electronic supplementary material

Supplementary information

Tables S1-4, figures S1, S2, S3
