# Supplementary material for: Proteo-metabolomics reveals compensation between ischemic and non-injured contralateral kidneys after reperfusion
Source: Sci Rep. 2018 Jun 4;8:8539. doi: 10.1038/s41598-018-26804-8 (PMC5986744; doi:10.1038/s41598-018-26804-8)
Supplement: Supplementary file 1 — Supplementary information [file 41598_2018_26804_MOESM1_ESM.pdf]

Proteo-metabolomics reveals compensation between ischemic and non-injured contralateral kidneys after reperfusion

Honglei Huang[1,2], Leon FA van Dullemen[3‡], Mohammed Z Akhtar[1‡], Maria-Letizia Lo Faro [1‡], Zhanru Yu[2‡], Alessandro Valli[2‡], Anthony Dona[4,5], Marie-Laëtitia Thézénas [2], Philip D Charles[2], Roman Fischer[2], Maria Kaisar[1,6], Henri GD Leuvenink[3], Rutger J Ploeg[1] and Benedikt M Kessler[2]

[1]Oxford Transplant Centre, Nuffield Department of Surgical Sciences, University of Oxford, UK
[2]Target Discovery Institute, Nuffield Department of Medicine, Old Road Campus, University of Oxford, UK
[3]Department of Surgery, University Medical Center Groningen, the Netherlands
[4]Department of Surgery and Cancer, Sir Alexander Fleming Building, Imperial College London, UK
[5]Cardiac Technology Centre, Kolling Institute of Medical Research, Royal North Shore Hospital, University of Sydney, Australia
[6]Research and Development Laboratory, NHS Blood and Transplant, Filton, UK

‡Authors contributed equally

SUPPLEMENTARY INFORMATION (methods, figure legends and tables)

1. Supplementary Methods
2. Supplementary Figures
   Figure S1 Histology and staining for apoptosis in endogenous controls
   Figure S2 Coagulation and complement pathway activation after IRI
   Figure S3 Mitochondrial complex II-III and IV activity analysis after IRI
3. Supplementary Tables
   Table S1 Differentially expressed proteins between 4h-IRI, 4h-C, and HC kidneys
   Table S2 Total of 363 differentially expressed proteins between 24h-IRI, 24h-C, and HC kidneys
   Table S3 Altered kidney metabolome after 4h IRI detected by NMR and GCxGC-MS
   Table S4 Proteins and metabolites used for integrated proteome and metabolome analysis

SUPPLEMENTARY INFORMATION

1. Supplementary Methods

Additional information on IRI animal model

A rat model of Ischaemia reperfusion injury was approved by the Animal Welfare Ethics Review Board, and carried out in accordance with Home Office guidance on Operation of Animals (PPL 30/2750). Adult male Fisher rats (F344) weighing 250 to 320g (Harlan, UK) were anaesthetized with isoflurane and placed on an auto-thermoregulatory heating pad. Continuous monitoring of oxygen saturation and heart rate was performed throughout the surgery. A midline laparotomy was performed with dissection of the right and left renal pedicles. After administration of heparin (10 units/ml) the left renal artery was clamped for 45min inducing warm ischaemia (IRI). Ischaemia was confirmed by visual inspection. The contralateral right kidney served as an endogenous control and remained untouched. During the warm ischaemic period the laparotomy incision was kept approximated and the rat maintained anaesthetized. After 45min the clamp was released and reperfusion of the left kidney was observed under visual inspection. The laparotomy incision was then closed and local anaesthetic was administered to the wound. Rodents were placed in a pre-warmed cage with free access to food and water. No rats were excluded from this study due to technical failure or death.

To evaluate short- and longer-term effects, after 4h and 24h reperfusion, respectively, rats were killed and both the left (IRI-4h; IRI-24h) and right (C-4h; C-24h) kidneys were retrieved for further analysis. As in this unilaterally applied IRI model, molecular injury afflicted to the left kidney may induce systemic effects, modulating the contralateral right kidney, we also included kidneys from healthy control (HC) rats as a baseline to discriminate between local injuries versus systemic effects. Procurement of all kidneys included cannulation of the aorta and flushing with cold saline. All kidney samples were either stored in formalin or snap frozen in liquid nitrogen to be kept at -80°C until use. Hence, the following groups of kidneys were included in this experiment: IRI-4h (n=7) and C-4h (n=7); IRI-24h (n=5) and C-24h (n=5); HC (n=4).

More information for protein extraction

The kidney cortex was dissected (20-30mg sections) and placed in beads-beater tubes containing lysis buffer (8M urea, 50mM Tris-HCL pH=8.5, 5mM DTT, 1% SDS, protease inhibitor) to make it 20mg/ml. Tissues were homogenized for 4 times at 6,500 Hz for 40s in a beads-beater (Stretton, UK). The samples were centrifuged at 10,000g for 5min at 4°C to remove insoluble tissue debris. The protein concentration in the homogenates was determined by BCA assay (Thermo Fisher, UK) and 100 µg of total proteins were added to a 30kDa filter (Merck Millipore, UK). Proteins were reduced by 20mM DTT (Sigma, UK) at 37°C for 1h, and then alkylated with 100mM iodoacetamide (IAA, Sigma, UK) for 45min in the dark, at room temperature. Samples were centrifuged for 20min at 14,000 g to remove DTT and IAA and followed by buffer exchange with 8 M urea twice and 50mM ammonia bicarbonate for 3 times. One hundred microliters of trypsin were added at a trypsin/protein ratio of 1:50 for digestion at 37°C overnight. Digested peptides were collected by upside down spin and membrane filters washed twice with 0.5M NaCl and water respectively. The peptides were purified by a SepPak C18 cartridge (Waters, UK), dried by Speed Vac centrifugation, and resuspended in buffer A (2% acetonitrile 0.1% formic acid) for LC-MS/MS analysis (Figure 1).

More information on protein identification and quantitation by mass spectrometry

LC-MS/MS analysis was carried out by Nano-ultra performance liquid chromatography tandem mass spectrometry analysis using a 75 µm-inner diameter x 25 cm C18 nanoAcquity UPLC column (1.7-µm particle size, Waters, UK). Peptides were separated with a 120min gradient of 3–40% solvent B (solvent A: 99.9% H_2O, 0.1% formic acid; solvent B: 99.9% ACN, 0.1% formic acid) at 250nl/min and injected into a Q Exactive High Field (HF) Hybrid Quadrupole-Orbitrap Mass Spectrometer (Thermo Scientific) acquiring data in electron spray ionisation (ESI) positive mode. The MS survey was set with a resolution of 60,000 FWHM with a recording window between 300 and 2,000 m/z. A maximum of 20 MS/MS scans were triggered in data-dependent acquisition (DDA) mode.

Analysis of mass spectrometry data

Raw LC-MS/MS data were uploaded to LC-Progenesis IQ software (Waters, UK), and total ion chromatograms (TICs) including retention time and m/z were used for automatic sample alignment. Merged files containing all the MS/MS spectra from all samples were submitted to a Mascot (V2.5) Matrix science search against the Swissprot rat database (UPR_Rattus Norvegicus, 34,165 entries). Proteins were identified with at least one matched peptide, a peptide score above 20, and an estimated false discovery rate (FDR) of 1%, representing confidence of identification. Protein quantities were measured by the sum of the top three unique peptide precursor ion intensities quantities, derived from the same protein after quantity normalization. Statistical analysis was conducted using one-way ANOVA for comparing the independent sample types. Alternatively, the in-house Central Proteome Facility Pipeline (CPFP) software was also used to identify peptides based on MS/MS spectra.[1] Three search engines including Mascot, OMSSA, and j were used for protein identification and combined with iProphet, with a FDR of 1%. The spectral index count (SINQ) was used for protein quantitation. Ingenuity pathway analysis (QIAGEN, UK) and KEGG (Kyoto Encyclopaedia of Genes and Genomes)[2] were used to assign differentially expressed proteins to pathways. PANTHER Classification system was used to perform gene ontology enrichment analysis (over-represented or under-represented). The mass spectrometry proteomics data have been deposited to the ProteomeXchange Consortium via the PRIDE partner repository with the dataset identifier PXD005101 and 10.6019/PXD005101.

More information on western blot validation of differentially expressed proteins

Ten milligram of renal cortex samples were dissected and lysed in 300µL of RIPA buffer (150mM NaCl, 1.0% NP-40, 0.5% sodium deoxycholate, 1% SDS, 50mM Tris, pH=8.0) containing a protease- (Roche, USA) and phosphatase inhibitor cocktail (Sigma, UK). Homogenization was performed on a beads beater at 6,500rpm for 40s. Protein concentration was quantified using BCA Protein (Thermo Fisher Scientific, UK). Samples containing 15µg of protein were denatured at 95°C for 5min in Laemmli buffer

and loaded onto 8–12% pre-cast SDS-PAGE gels (Bio-Rad, USA). Proteins separated by SDS-PAGE were transferred to hydrophobic PVDF membranes (Merck Millipore, USA) in transfer buffer (25mM Tris, 192mM glycine and 10% methanol) overnight. PVDF membranes were blocked for 1h in TBST buffer (25mM Tris, pH=7.5, 0.15 M NaCl, 0.05% Tween 20) containing 5% milk. Membranes were incubated overnight at 4 °C with rabbit anti-rat C4 (25ug/ml), HSP70 (1:1000), GST (1:3000), FABP4 (1mg/ml), BVR (1:5000) (Abcam, UK), and TLR4 (1:250) (Santa Cruz) antibodies. Mouse monoclonal anti β-actin was used as loading control (1:25,000) (Sigma, Germany). Membranes were washed for 30min with 5 changes of wash buffer and then incubated at room temperature for 1h in blocking buffer containing a 1:5000 dilution of Dye-800-conjugated anti-mouse or -rabbit secondary antibody (Li-Cor, USA), and visualization was performed with Odyssey CLx (Li-Cor, USA). Detected signals were quantified and normalized to the β-actin signal on the same blot using ImageJ (1.47v, USA). Difference in mean was calculated with a Mann-Whitney U test (Prism v5.0, GraphPad Software, USA). Significance was set at a value of $p<0.05$ and all graphs report results as mean ± standard error of the mean.

More information on extraction of metabolites from kidney tissue

Fifty micrograms of kidney cortex tissues were weighed and placed in beads-beater tubes containing 1.5ml of pre-chilled methanol/water (1:1). The tissues were frozen on dry ice (around 2min) and then loaded onto a beads beater (Stretton, UK). Tissues were homogenised four times at 6,500Hz for 40s and cooled on dry ice for 2min between cycles. Homogenates were centrifuged at 13,000g for 20min at 4°C. Supernatants (aqueous fractions) were transferred to an Eppendorf tube, dried in a Speed Vac and stored at -80°C until use. For organic extraction, 1.6ml of pre-chilled dichloromethane/methanol (3:1) was added to the tissue pellet and chilled on dry ice for 2min. Pellets were further homogenized as previously described, and centrifuged at 13,000g for 20min. Supernatants were collected into glass vials. The organic extraction was dried in a fume cupboard overnight with lids open. The extracted samples were stored at -80°C until analysis (Figure 1).

Metabolomic analysis using nuclear magnetic resonance spectroscopy (NMR)

Sample preparation and acquisition methods by ^{1H}NMR were adapted from previously published methods.[3,4] In brief, extracted aqueous kidney pellets were resuspended before NMR acquisition in 550µl phosphate buffer (0.2M Na_2HPO_4/0.04 M NaH_2PO_4, pH=7.4 with 0.1% sodium azide and 1mM 3-trimethylsilyl-1-[2,2,3,3,-2H4] propionate (TSP) in D_2O), vortexed for 2min, and then transferred to a 5 mm NMR tube for analysis. ^{1H}NMR spectra were acquired with a Bruker Avance 600MHz nuclear magnetic resonance spectrometer instrument operating at 600.13MHz for 1h at 300K. Tissue extracts were analysed using water suppressed ^{1D}NMR spectrum using the NOESYPRESAT pulse sequence (256 transients). Irradiation of the solvent (water) resonance was applied during pre-saturation delay (2.0s) for all spectra and for the water suppressed ^{1D}NMR spectra also during the mixing time (0.1s). Pulse sequence parameters including the 90° pulse (~ 12µs), pulse frequency offset (~ 2,800Hz), receiver gain (~ 200), and pulse powers were optimised. The spectral width was 20ppm for all spectra. NMR data was processed with an exponential line broadening of 1.0Hz prior to Fourier transformation, which was collected with approximately 32k real data points. For NMR spectral data pre-processing, data sets [-1.0 to 10.0ppm] were imported into MATLAB 7.0 software (MathWorks, USA), where they were automatically phased, baseline corrected and referenced to the TSP peak (0.00 ppm) using scripts written in-house. To reduce analytical variation between samples the residual water signal (4.67 – 4.98ppm) was truncated from the data set. Normalization using probabilistic quotient (median fold change) methods was then performed.[5] Assignments of endogenous metabolites from kidney tissue were made by reference to published literature data[6] as well as with use of the in-house and online databases.[7,8] Following the processing of the NMR data, multivariate statistical analysis was performed using SIMCA-P 13.0. Principle Component Analysis (PCA) and Orthogonal Partial-Least Squares Discriminant analysis (OPLS-DA) using both univariate and mean centred scaling were used to identify specific metabolites pertaining to a particular sample group.[9] All OPLS models were run through random permutation testing to assess the validity of the supervised model.

Metabolomic analysis using gas chromatography mass spectrometry (GCxGC-MS)

For chemical derivatisation, extracted organic fractions from kidney cortex were oximated at 30°C for 90min using 20mg/ml of methoxyamine hydrochloride (Sigma) dissolved in pyridine. Subsequently, samples were silylated at 60°C for 60min using *N*-methyl-*N*-trimethysilyltrifluroacetamide (MSTFA). Deuterium labelled myristic acid was spiked into tissue (5ml/10mg of wet tissue) to serve as an internal standard for data normalization. Two ml of derivatised metabolites were injected into a GCxGC-qMS instrument (GP2010, Shimadzu) with a split ratio of 1 in 20 in pyridine. The oven temperature was programmed to rise from 80°C to 330°C at a rate of 6°C per min, and the total run time was 35 min. The metabolites were first separated by a non-polar column (30m x 0.25mm i.d), followed by a second polar column (6m x 0.25mm i.d) controlled by a modulator that operated at six second cycles as described.[10] In brief, metabolite effluents separated on the first column were accumulated for 6s, then remobilized, focused and injected into the second column. Ions were generated within GC-MS by electron impact (EI) ionization and collected at a mass range of 50*m/z* to 800*m/z* using an acquisition rate of 20,000u/sec and 100Hz. Metabolites were identified by matching collected fragment ions to the NIST library using GC solution software (Ver.2.32). The identification confidence score cut off was set to 80, and a panel of collected standards were used to verify their identification (such as palmitic acid, stearic acid). Metabolites quantitation was performed using Chrome square software 2.1.6 (Shimadzu, Japan). Each metabolite quantity was normalized to spiked myristic acid for comparison.

Luminescent determination of ATP concentrations in the kidney cortex

ATP was extracted according to protocol[11] and concentrations were measured using ENLITEN ATP assay kits (Promega, UK) and FLUOstar Omega (BMG LABTECH, UK) plate reader. In brief, 40mg of kidney tissue was homogenised in 1ml ice cold Tris-EDTA (10mM, pH 8.0, 1mM EDTA-saturated phenol (phenol-TE) using an Ultra Turrax homogeniser. Homogenate was transferred to micro-tubes containing 200µl chloroform and 150µl water. After mixing and centrifugation at 10,000xg for 5min at 4°C, the aliquot was diluted 100 times for luciferin-luciferase analysis. The assay was performed by

adding 0.1ml of the reagent per well and read on a microplate reader. The assay was performed by adding 90µl of reagent per well to 10µl of samples and read on a microplate reader. ATP was measured by comparing to a standard curve after correction against blank samples. A Kruskal-Wallis (non-parametric) comparison of multiple groups was performed (Prism v5.0, GraphPad Software, USA). Significance was set at a value of $p<0.05$ and all graphs report results as mean±SD.

Respiratory control ratio (RCR) measurement after 24h IRI

Mitochondria were isolated from fresh kidney tissues following Frezza's protocol.[12] Mitochondrial complex activity assays were performed on a microplate reader (BMG Labtech).[13] Complex activity was normalized to citrate synthase activity .[12] For the oxygen consumption assays, fresh kidney tissues were used for mitochondrial isolation. State I respiration was recorded with the addition of 1mg/ml of mitochondrial suspension to the Clark electrode. State II and -III respirations were recorded with the addition of 5mM succinate and 100µM ADP respectively. State IV was measured following ADP consumption from the solution and state V was induced by adding 60nM of carbonyl cyanide p-(tri-fluromethoxy) phenyl-hydrazone (FCCP). The respiratory control ratio (RCR) was calculated by dividing state III by state IV respiration.[12] Statistical significance was calculated using Mann-Whitney U test and set at p-value <0.05.

More information about integrative analysis of proteome and metabolome data

To provide a global view of the proteo-metabolome data, a knowledge-based targeted proteome and metabolome data integration approach was applied making use of the human protein sequence database UniProtKB and the human metabolome database (HMDB).[14-16] Based on altered metabolome profiles between 4h-IRI, 4h-C, and HC kidneys, all the proteins/enzymes involved in energy metabolism, such as glycolysis, TCA cycle, FA transporter, FA β-oxidation and mitochondrial respiration chain for ATP metabolism were shortlisted. Proteins regulated by metabolites (Ampd2) that were changed in IRI were also included from the proteome data. A total of 125 proteins were

selected in the analysis, from which 29 were shortlisted based on their statistical significance and altered abundance (Table S4). From all measured and quantified 71 metabolites, we selected the subset (16) that changed in a significant manner. A non-parametric Mann-Whitney test was applied to probe the significance of differences observed for both proteins and metabolites. We then used R-based clustering of proteins and metabolites to visualise subgroups with different patterns of abundance (Figure 8).

2. Supplementary Figures

Figure S1. Histology and staining for apoptosis in cortex and medulla sections of endogenous controls.

A. Periodic Acid-Schiff (PAS) staining for histological changes in one selected contralateral kidney per condition (4h-C and 24h-C) and healthy controls (HC). Formalin fixed kidney tissue from contralateral kidneys were used for PAS staining. No histological changes were evident after 4h or 24h reperfusion in the contralateral kidneys. **B.** Terminal Deoxynucleotidyl Transferase dUTP nick end labelling (TUNEL) staining for apoptosis in contralateral kidneys (4h-C and 24h-C) and healthy controls (HC). Breakdown of DNA is shown in red, while nuclei were stained blue with DAPI. Co localization of GFP and DAPI is shown as pink. No apoptotic tubular cells were detected at 4h-C or 24h-C in both cortex and medulla sections.

Figure S2. Coagulation and complement pathway activation after IRI.

A. KEGG pathway mapping (www.kegg.jp/kegg/kegg1.html) shows upregulation of differentially expressed proteins involved in the coagulation and complement pathway after 4h-IRI and 24h-IRI. Most proteins mapped to this pathway were significantly (≥2-fold) upregulated in 24h-IRI as compared to 24h-C, however, the changes in 4h-IRI vs 4h-C were mostly indifferent and not significant as compared to 4h-C. **B.** There was an increased level of C4 in both 4h-IRI and 24h-IRI measured by Western blot.

Data is presented as mean±SEM. 4h-IRI and 24h-IRI: kidneys subjected to IRI; 4h-C and 24h-C: contralateral controls; HC: healthy controls.

Figure S3. Mitochondrial complex II-III and IV activity analysis after IRI.

A. Complex II-III activity was measured after 4h and 24h post IRI in injured (4h-IRI, 24h-IRI), contralateral (4h-C, 24h-C), and healthy controls (HC). **B.** Normalized complex II-III activity after

normalization to citrate synthase. **C.** Complex IV activity was measured using the same set of samples as A. **D.** Normalized complex IV activity after normalization to citrate synthase. No statistical significant changes were detected between the different groups.

Data is expressed as mean±SD.

3. Supplementary Tables

Table S1 **Differentially expressed proteins between 4h-IRI, 4h-C, and HC kidneys**

Table S2 **Total of 363 differentially expressed proteins between 24h-IRI, 24h-C, and HC kidneys**

Table S3 **Altered kidney metabolome after 4h IRI detected by NMR and GCxGC-MS**

Table S4 **Proteins and metabolites used for integrated proteome and metabolome analysis**

References

1 Trudgian, D. C. *et al.* CPFP: a central proteomics facilities pipeline. *Bioinformatics* **26**, 1131-1132, doi:10.1093/bioinformatics/btq081 (2010).

2 Kanehisa, M. & Goto, S. KEGG: kyoto encyclopedia of genes and genomes. *Nucleic acids research* **28**, 27-30 (2000).

3 Beckonert, O. *et al.* Metabolic profiling, metabolomic and metabonomic procedures for NMR spectroscopy of urine, plasma, serum and tissue extracts. *Nature protocols* **2**, 2692-2703, doi:10.1038/nprot.2007.376 (2007).

4 Dona, A. C. *et al.* Precision high-throughput proton NMR spectroscopy of human urine, serum, and plasma for large-scale metabolic phenotyping. *Analytical chemistry* **86**, 9887-9894, doi:10.1021/ac5025039 (2014).

5 Craig, A., Cloarec, O., Holmes, E., Nicholson, J. K. & Lindon, J. C. Scaling and normalization effects in NMR spectroscopic metabonomic data sets. *Analytical chemistry* **78**, 2262-2267, doi:10.1021/ac0519312 (2006).

6 Bollard, M. E., Stanley, E. G., Lindon, J. C., Nicholson, J. K. & Holmes, E. NMR-based metabonomic approaches for evaluating physiological influences on biofluid composition. *NMR in biomedicine* **18**, 143-162, doi:10.1002/nbm.935 (2005).

7 Ichas, F. & Mazat, J. P. From calcium signaling to cell death: two conformations for the mitochondrial permeability transition pore. Switching from low- to high-conductance state. *Biochimica et biophysica acta* **1366**, 33-50 (1998).

8 Wishart, D. S. *et al.* HMDB: the Human Metabolome Database. *Nucleic acids research* **35**, D521-526, doi:10.1093/nar/gkl923 (2007).

9 Barton, R. H., Nicholson, J. K., Elliott, P. & Holmes, E. High-throughput 1H NMR-based metabolic analysis of human serum and urine for large-scale epidemiological studies: validation study. *International journal of epidemiology* **37 Suppl 1**, i31-40, doi:10.1093/ije/dym284 (2008).

10 Beens, J., Adahchour, M., Vreuls, R. J., van Altena, K. & Brinkman, U. A. Simple, non-moving modulation interface for comprehensive two-dimensional gas chromatography. *Journal of chromatography. A* **919**, 127-132 (2001).

11 Chida, J., Yamane, K., Takei, T. & Kido, H. An efficient extraction method for quantitation of adenosine triphosphate in mammalian tissues and cells. *Analytica chimica acta* **727**, 8-12, doi:10.1016/j.aca.2012.03.022 (2012).

12 Frezza, C., Cipolat, S. & Scorrano, L. Organelle isolation: functional mitochondria from mouse liver, muscle and cultured fibroblasts. *Nature protocols* **2**, 287-295, doi:10.1038/nprot.2006.478 (2007).

13 Kiebish, M. A. *et al.* Lipidomic analysis and electron transport chain activities in C57BL/6J mouse brain mitochondria. *Journal of neurochemistry* **106**, 299-312, doi:10.1111/j.1471-4159.2008.05383.x (2008).

14 May, P., Christian, N., Ebenhoh, O., Weckwerth, W. & Walther, D. Integration of proteomic and metabolomic profiling as well as metabolic modeling for the functional analysis of metabolic networks. *Methods Mol Biol* **694**, 341-363, doi:10.1007/978-1-60761-977-2_21 (2011).

15 Kosieradzki, M. & Rowinski, W. Ischemia/reperfusion injury in kidney transplantation: mechanisms and prevention. *Transplantation proceedings* **40**, 3279-3288, doi:10.1016/j.transproceed.2008.10.004 (2008).

16 Valli, A. *et al.* Hypoxia induces a lipogenic cancer cell phenotype via HIF1alpha-dependent and -independent pathways. *Oncotarget* **6**, 1920-1941, doi:10.18632/oncotarget.3058 (2015).

Figure S1

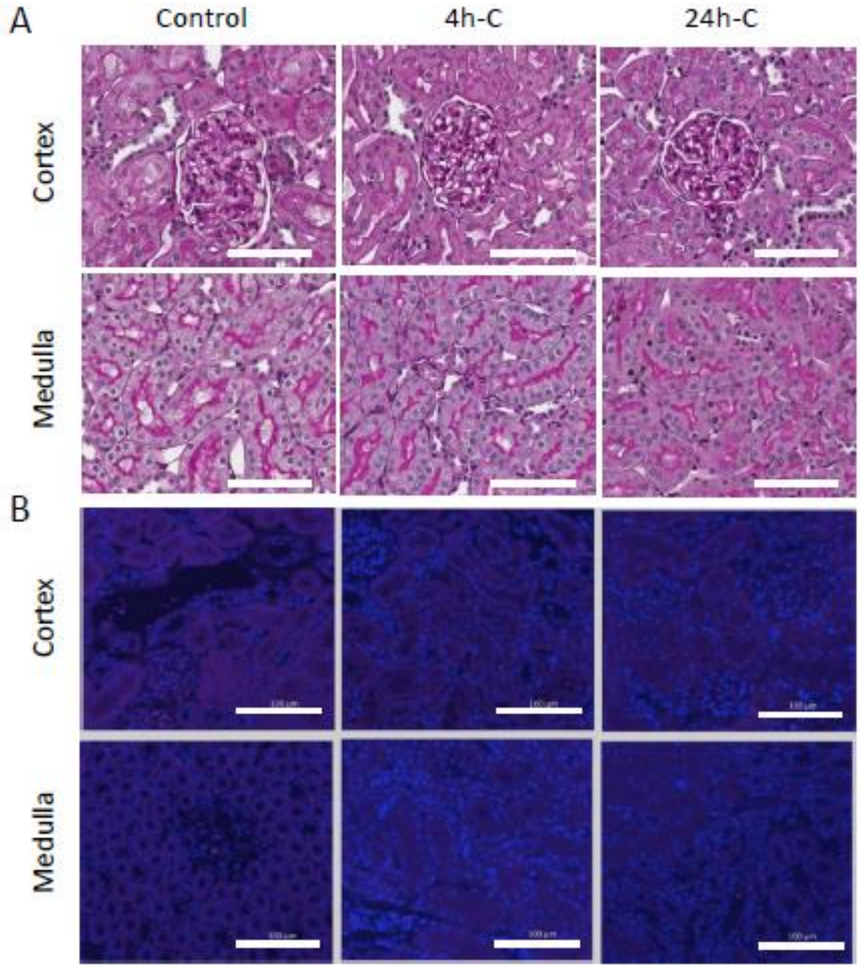

Figure S2

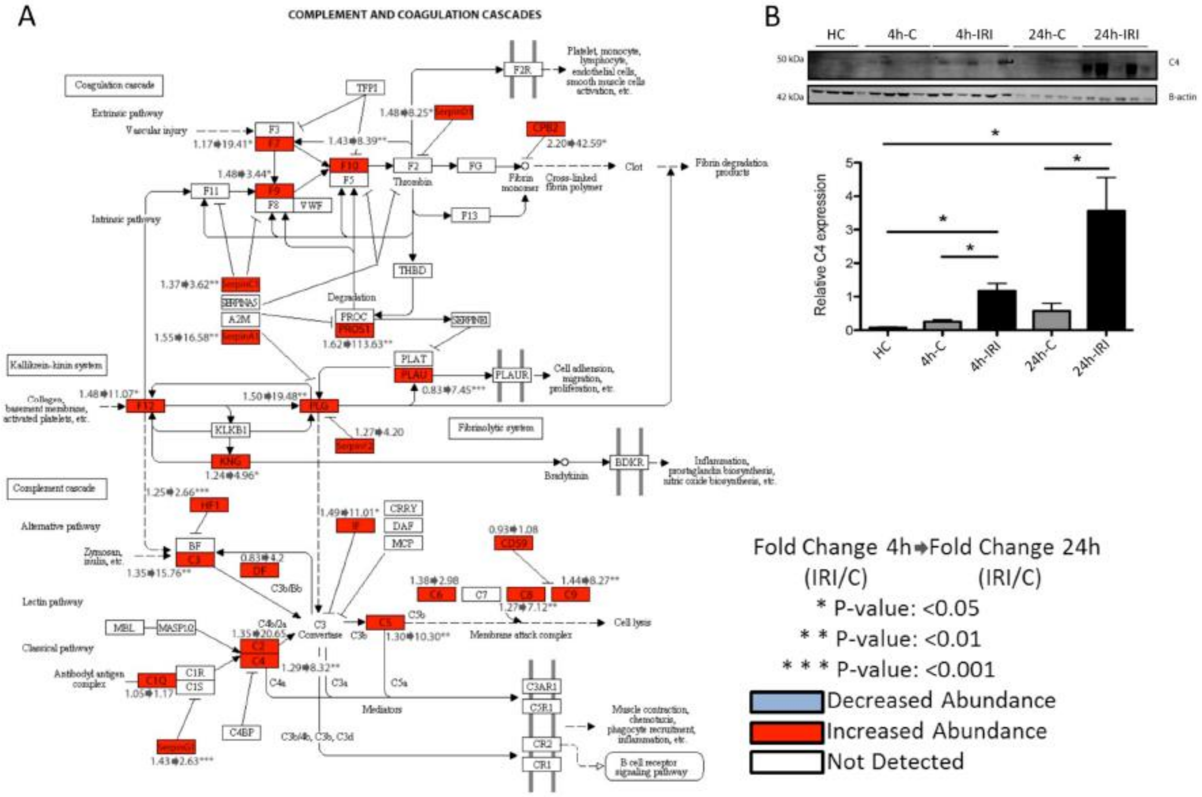

A
COMPLEMENT AND COAGULATION CASCADES
B
HC
4h-C
4h-IRI
24h-C
24h-IRI
C4
B-actin
Relative C4 expression
Fold Change 4h➡Fold Change 24h
(IRI/C) (IRI/C)
* P-value: <0.05
* * P-value: <0.01
* * * P-value: <0.001
Decreased Abundance
Increased Abundance
Not Detected

Figure S3

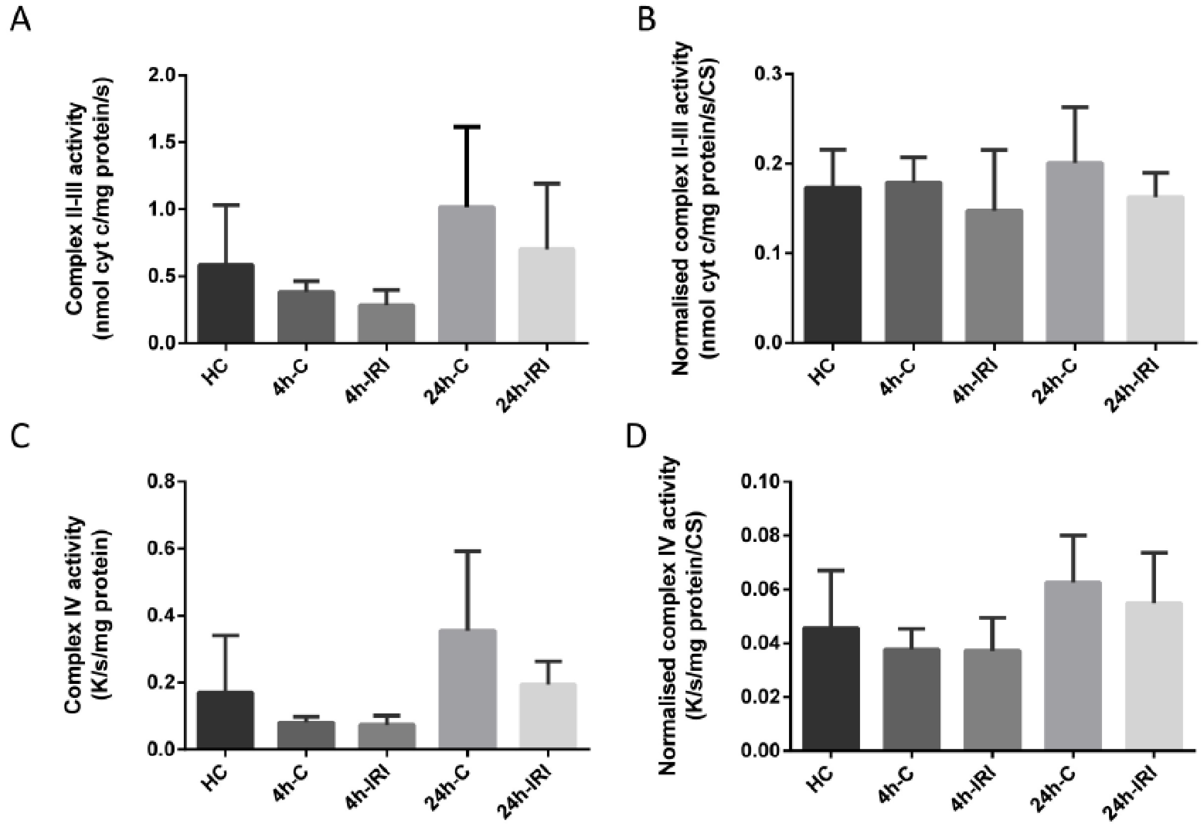
A
Complex II-III activity (nmol cyt c/mg protein/s)
2.0
1.5
1.0
0.5
0.0
HC
4h-C
4h-IRI
24h-C
24h-IRI
B
Normalised complex II-III activity (nmol cyt c/mg protein/s/CS)
0.3
0.2
0.1
0.0
HC
4h-C
4h-IRI
24h-C
24h-IRI
C
Complex IV activity (K/s/mg protein)
0.8
0.6
0.4
0.2
0.0
HC
4h-C
4h-IRI
24h-C
24h-IRI
D
Normalised complex IV activity (K/s/mg protein/CS)
0.10
0.08
0.06
0.04
0.02
0.00
HC
4h-C
4h-IRI
24h-C
24h-IRI
